# Comprehensive Study of Atorvastatin Nanostructured Lipid Carriers through Multivariate Conceptualization and Optimization

**DOI:** 10.3390/pharmaceutics13020178

**Published:** 2021-01-28

**Authors:** Heba A. Ghanem, Ali M. Nasr, Tamer H. Hassan, Mahmoud M. Elkhoudary, Reem Alshaman, Abdullah Alattar, Shadeed Gad

**Affiliations:** 1 Department of Pharmaceutics, Faculty of Pharmacy, Sinai University, Al Qantarah Sharq 41636, Egypt; heba.awd@su.edu.eg; 2 Department of Pharmaceutics, Faculty of Pharmacy, Port Said University, Port Said 42526, Egypt; 3 Department of Pharmaceutics, Faculty of Pharmacy, Sinai University, North Sinai 45511, Egypt; 4 Department of Pharmaceutics, Faculty of Pharmacy, Suez Canal University, Ismailia 41522, Egypt; shaded_abdelrahman@pharm.suez.edu.eg; 5 Department of Pharmaceutical Chemistry, Faculty of Pharmacy, Horus University-Egypt, New Damietta 34518, Egypt; melkhodary@horus.edu.eg; 6 Department of Pharmacology and Toxicology, Faculty of Pharmacy, University of Tabuk, Tabuk 471, Saudi Arabia; ralshaman@ut.edu.sa (R.A.); aalattar@ut.edu.sa (A.A.)

**Keywords:** hyperlipidemia, atorvastatin, nano-structured lipid carriers, combined D-optimal screening design, central composite design

## Abstract

The aim of the current study is to establish a comprehensive experimental design for the screening and optimization of Atorvastatin-loaded nanostructured lipid carriers (AT-NLCs). Initially, combined D-optimal screening design was applied to find the most significant factors affecting AT-NLCs properties. The studied variables included mixtures of solid and liquid lipids, the solid/liquid lipid ratio, surfactant type and concentration, homogenization speed as well as sonication time. Then, the variables homogenization speed (A), the ratio of solid lipid/liquid lipid (B), and concentration of the surfactant (C) were optimized using a central composite design. Particle size, polydispersity index, zeta potential, and entrapment efficiency were chosen as dependent responses. The optimized AT-NLCs demonstrated a nanometric size (83.80 ± 1.13 nm), Polydispersity Index (0.38 ± 0.02), surface charge (−29.65 ± 0.65 mV), and high drug incorporation (93.1 ± 0.04%). Fourier Transform Infrared Spectroscopy (FTIR) analysis showed no chemical interaction between Atorvastatin and the lipid mixture. Differential Scanning Calorimetry (DSC) analysis of the AT-NLCs suggested the transformation of Atorvastatin crystal into an amorphous state. Administration of the optimized AT-NLCs led to a significant reduction (*p* < 0.001) in serum levels of rats’ total cholesterol, triglycerides, and low-density lipoproteins. This change was histologically validated by reducing the relevant steatosis of the liver.

## 1. Introduction

Hyperlipidemia represents one of the most widespread metabolic disorder diseases. It is distinguished by elevated levels of serum total cholesterol (TC), triglycerides (TG), and low-density lipoprotein cholesterol (LDL) associated with a decrease in the concentration of serum high-density lipoprotein cholesterol (HDL) [1]. It comprises one of the key risk factors for cardiovascular and cerebral diseases such as atherosclerosis, coronary artery diseases, myocardial infarction, stroke as well as cardiac sudden deaths [2,3]. Preventing hyperlipidemia is crucial for the prevention of cerebral and cardiovascular diseases [4]. Atorvastatin (AT) belongs to a drug class, known as statins, that lower the blood cholesterol level. It is a 3-hydroxy-3-methylglutaryl coenzyme A (HMG-CoA) reductase competitive inhibitor. This enzyme catalyzes the conversion of HMG-CoA to mevalonate, a rate-limiting step in cholesterol biogenesis [5]. AT bioavailability is approximately 12% and is classified as a class II drug according to the Biopharmaceutical Classification System (BCS) [6]. Lipid-based formulations have emerged as excellent carriers for oral formulations due to the diverse physicochemical properties of lipids, their biocompatibility, and lymphatic uptake, especially for drugs suffering from first-pass effect and incomplete intestinal absorption [7]. Several lipid-based formulations have been used to improve the bioavailability of AT such as self-emulsifying drug delivery systems (SEDDS) [8], a self-nano emulsifying drug delivery system (SNEDDS) [9], solid lipid nanoparticles (SLNs) [10], and nanocrystals [11]. Nanostructured lipid carriers (NLCs) are second-generation solid lipid nanoparticles (SLNs) that combine the advantages of the SLN with high entrapment efficiency and reduced drug leakage throughout storage [12]. NLCs were previously used to enhance the oral bioavailability of different drugs [13,14,15]. Atorvastatin-loaded NLCs (AT-NLCs) were reported earlier to improve oral AT bioavailability [16,17]. However, there was no comprehensive study about the most significant factors affecting their properties and pharmacodynamics. Quality by Design (QbD) is a multivariate experimental design that focuses on the control strategy and robustness. The QbD experimental designs are considered to be a pivotal method providing maximum information using minimal experimentation [18]. The application of the Design of Experiment (DoE) would be helpful to understand the relationship between independent factors and their responses in drug formulations. The influence of each formulation variable as well as the interaction between different independent factors can be analyzed using DoE in order to assess the most critical variables [19]. By and large, DoE has three major areas in drug formulations: initially, the screening phase that identifies the most critical factors for a certain process and their appropriate levels [19]. Then, the optimization phase that detects the optimum input variables with their levels to obtain the best possible responses [20]. Finally, to determine the sensitivity of response to small changes in the factors. This study aims to understand the most significant formulation variables affecting AT-NLC properties such as particle size, polydispersity index, zeta potential, and entrapment efficiency using the capabilities of the D-optimal design, and furthermore, to optimize the most significant variables to achieve AT-NLC formulation with appropriate properties using the proficiencies of the central composite design. Finally, the optimized AT-NLC formulation was characterized for physicochemical properties, in-vitro release, pharmacodynamics as well as suitable histopathology examination.

## 2. Materials and Methods

### 2.1. Materials

Atorvastatin was kindly gifted from Al-Arabiya Pharmaceutical Company (Cairo, Egypt). Stearic Acid was obtained from Hi-Media Laboratories Pvt. L-td. (Mumbai, India). Labrasol (PEG-8 caprylic/capric glycerides), Capryol PGMC (propylene glycol monocaprylate type I), Labrafac PG (propylene glycol dicaprylate/dicaprate), Compritol ATO 888 (glycerol dibehenate/behenate), Compritol ATO 888 CG, and Gelucire 43/01(mixtures of mono-, di- and triglycerides with PEG esters of fatty acids) were kindly gifted from Gattefosse (Saint-Priest, France). Beeswax was purchased from Alexandria mineral oil company (Alexandria, Egypt). Tween 80 (polyoxyethylen-80-sorbitanmonooleate, polysorbate 80) was purchased from ADWIC Chemicals Co. Cairo, Egypt. Poloxamer 188 was purchased from Sigma Aldrich, (St. Louis, MO, USA). Pure soybean lecithin was purchased from the DASIT company (Val de Reuil, France). All reagents were of analytical grade and were used without further purification.

### 2.2. Preliminary Study

#### 2.2.1. Partitioning Behavior of AT in Solid Lipids

AT (10 mg) was mixed with 1 g of the solid lipid (Beeswax, Compritol ATO 888 CG, Stearic acid, Compritol ATO 888, and Gelucire 43/01). The solid lipid and 3 mL of distilled water were heated together above 70 °C in a water bath. Afterwards, the melted mixture was mixed thoroughly by a vortex mixer (MaxiMix II, Orlando, FL, USA). By repeated exposure of the melted lipid to the heated water bath and vortex mixer, solidification was prevented. The mixture was centrifuged at 15,000 rpm for 30 min at 25 ± 0.5 °C by a centrifuge (model 2-16P, Sigma, Osterode am Harz, Germany) to separate the lipid. The resultant supernatant (aqueous phase) was diluted in order to quantify the amount of AT using a UV spectrophotometer (Tokyo, Japan) [21] The partition coefficient (Log P) was then calculated using the following equation [22]:

(1) $$\mathrm{PC}=\log\frac{\mathrm{Added}\;\mathrm{drug}\;\mathrm{content}-\mathrm{Drug}\;\mathrm{content}\;\mathrm{in}\;\mathrm{aqueous}\;\mathrm{phase}}{\mathrm{Drug}\;\mathrm{content}\;\mathrm{in}\;\mathrm{aqueous}\;\mathrm{phase}}$$

#### 2.2.2. Equilibrium Solubility of AT in Different Oils

The solubility of AT in different oils (Labrasol, Labrafac PG, Oleic acid, and Capryol PGMC) was determined by adding an excess amount of AT to 2 mL of the oil in a centrifuge tube. The tubes were mixed using a vortex mixer. An isothermal mechanical shaker (Clifton shaking water bath, London, UK) operated at 25 ± 0.5 °C was used to continuously agitate the centrifuge tubes for 72 h in order to reach equilibrium. Samples were transferred from the shaker to a centrifugation machine and centrifuged at 15,000 rpm for 30 min. To filter the supernatant, a 0.22 μm membrane filter (HiMedia, Mumbai, India) was used [21]. The filtered supernatant was assayed by a UV spectrophotometer.

### 2.3. Preparation of AT-NLCs

AT-NLCs were prepared using a high-shear homogenization method associated with sonication. First, the lipid phase was prepared by heating solid lipids (Stearic acid or Gelucire 43/01 or their combinations) at 10 °C above their melting points by a heating magnetic stirrer (Brandstead/Thermolyne, Swedesboro, NJ, USA). The liquid lipid (Labrasol or oleic acid or their combinations) was added to the solid lipid. AT (with or without lecithin) was added to the lipid phase. The aqueous phase, which contained surfactant (Tween 80 or Poloxamer 188) and bidistilled water, was heated at the same temperature of the lipid phase separately. After reaching the same temperature, the heated aqueous phase was added dropwise to the molten lipid phase and mixed at 600 rpm for 10 min by the heating magnetic stirrer. Subsequently, the mixtures were homogenized using a Heidolph Silent Crusher^®^ homogenizer (Heidolph, Schwabach, Germany) for 10 min. Finally, the formulations were subjected to a digital sonifier (Branson, Danbury, CT, USA) for 5–10 min [23]. All formulations were kept in tightly closed containers for further investigations.

### 2.4. Experimental Designs

#### 2.4.1. Combined D-optimal Design

Preliminary screening was conducted to identify the most significant variables that may influence AT-NLC preparation. A combined D-optimal design was chosen as the screening design of experiments (DOE). The model design combined numeric, categoric as well as mixed independent variables that encompass two levels and eleven factors with 46 total runs. Dependent variables were the zeta potential, mean particle size, and polydispersity index (PDI). Data analysis was performed using Design-Expert^®^ version 11 software (Stat-Ease Inc., Minneapolis, MN, USA). A summary of the studied variables and their levels is presented in Table 1.

#### 2.4.2. Central Composite Design

Central Composite Design (CCD) was used to optimize the most significant variables that originated from the previous D-optimal design screening. This led to an optimized formulation with the best size, PDI, zeta potential, and entrapment efficiency. CCD with three independent variables, five levels, and 20 runs was performed using Design-Expert^®^ version 11 software. The independent variables were A: Homogenization speed; B: Solid/Liquid ratio, and C: Surfactant concentration. Dependent variables were particle size (Y1), PDI (Y2), zeta potential (Y3), and entrapment efficiency (EE%) (Y4). The objectives were to minimize the particle size (Y1) and polydispersity index (PDI) (Y2), to obtain a zeta potential value (Y3) from −35 mV to −25 mV, and to maximize the entrapment efficiency (EE%) (Y4).

### 2.5. Particle Size (PS) and Zeta Potential (ZP)

The AT-NLC average particle size (expressed as the Z-average), polydispersity index (PDI), and zeta potential (ZP) were accurately measured using a Zetasizer (Malvern Instruments Ltd., Malvern, UK) at 25 °C. All measurements were performed in triplicate and are expressed as the average ± standard deviation (SD). Each sample was diluted 20 times with an appropriate amount of distilled water before the analysis [24].

### 2.6. Entrapment Efficiency (EE%)

The EE% of AT-NLC dispersion was assessed by the centrifugation method [25]. A known volume of freshly prepared AT-NLCs was centrifuged at 12,000 rpm and 4 °C for 1 h using a cooling centrifuge (2-16KL, Sigma Laborzentrifugen GmbH, Osterode am Harz, Germany). The supernatant was diluted to a suitable concentration, and the concentration of the free (non-entrapped) drug was measured spectrophotometrically against a blank at 246 nm. AT entrapment efficiency was then determined using the following equation [26]:

(2) $$\;\mathrm{EE}\%=\frac{W_{t}\;-{\;W}_{f}}{W_{t}}\times 100$$

where EE% is the entrapment efficiency, W_t_ is the amount of added drug during the preparation of NLCs, and W_f_ is the free drug amount in the supernatant after centrifugation.

### 2.7. Differential Scanning Calorimetry (DSC) 

Pure AT powder, physical mixture, lyophilized AT-NLCs (optimized formulation), and lyophilized blank NLCs were investigated using a differential scanning calorimeter (DSC 6000; Perkin Elmer, Waltham, MA, USA). The ratio between AT and the excipient in the physical mixture was the same as the optimized AT-NLC formulation. Reasonable sample amounts (2–5 mg) were sealed hermetically in an aluminum pan and heated at a rate of 10 °C/min from 20 °C to 220 °C using another empty aluminum pan as a control [27].

### 2.8. Fourier Transform Infrared Spectroscopy (FTIR)

FTIR spectra of pure AT, blank NLC, the optimized AT-NLCs, and physical mixture were generated using the potassium bromide press method. Samples were mixed with dry infrared (IR)-grade crystalline potassium bromide and then compressed at 10 tons to form a thin disk by a hydraulic press. The recorded spectra were measured in the range of 400 to 4000 cm^−1^ using a Fourier transform infrared spectrophotometer (FTIR-4100 Jasco, Tokyo, Japan) [28].

### 2.9. Transmission Electron Microscopy (TEM)

The optimized AT-NLC formulation was visualized by TEM for the determination of the surface morphology. Samples were diluted 50 times with double distilled water and were negatively stained with phosphotungstic acid and then dried on a carbon-coated grid. A transmission electron microscope (JTEM model 1010, JEOL^®^, Tokyo, Japan) operated at 100 kV was used to visualize the samples [29].

### 2.10. In Vitro Release Studies

Release studies were carried out to assess the pattern of the in vitro drug release using the dialysis bag technique [30]. The dialysis bag with a molecular weight cutoff of 12 kDa (SERVAPOR^®^ Dialysis Membranes, Heidelberg, Germany) was soaked overnight before use in the release medium (phosphate buffer pH = 6.8). One mL of the optimized AT-NLC formula was added to the dialysis bag and immersed in 100 mL of preheated buffer in beakers. The beakers were incubated at 37 °C and their contents were rotated at about 100 rpm in a thermostatic shaker. At predefined time intervals of 0.5, 1, 2, 4, 8, 12, 24, and 48 h, dissolution medium aliquots were withdrawn for UV analysis at 247 nm. The experiments were done in triplicate against a blank. The release data were fitted to a zero-order release kinetic model, first-order release kinetic model, the Higuchi diffusion model, and the Hixson–Crowell model to investigate the AT release kinetics [31].

### 2.11. In-Vivo Study

#### 2.11.1. Experimental Animals

The research protocol was reviewed and accepted by Suez Canal University’s Institutional Animal Ethics Committee (Approval number; 2020 RA1). Male Wister rats (130–140 g) aged six weeks were obtained from the Animal House at the Faculty of Medicine, Suez Canal University. One week before the experiment, the rats were allowed to acclimate to the experimental humidity and temperature conditions. The rats were routinely fed with a diet of rat pellets and were allowed free access to water. The rats were held throughout the study at a temperature of 25 ± 2 °C with 12 h of dark/light cycles. 

#### 2.11.2. Pharmacodynamics Study

Sixteen male albino rats (300 ± 50 g) were fed with a high-fat diet. Commercial rat chow (75.5%), egg yolk (12.5%), lard (8.5%), cholesterol (3%), and bile salt (0.5%) were mixed, pelleted, and provided to Wistar rats for four weeks to initiate hyperlipidemia. Additionally, four animals were fed with a normal diet to serve as a negative control. Blood samples were collected from the caudal vein of animals and evaluated for serum lipid levels [11]. A considerable elevation in serum lipid levels indicated the establishment of hyperlipidemia with the high-fat diet. All 20 animals were randomly segregated into five groups (*n* = 4). Animals in each group were treated with respective regimens consecutively for two weeks after the induction of hyperlipidemia (except group I treated with normal saline) [32]. The groups were classified as follows:

- Group I: The negative control group that had no hyperlipidemia.
- Group II: The positive control group that was orally treated with 0.5% carboxymethyl cellulose sodium solution.
- Group III: The AT suspension-treated group that received oral treatment (25 mg/kg) with pure AT suspension.
- Group IV: The Lipitor^®^ 20-treated group that received oral treatment with Lipitor^®^ 20 (equivalent to 25 mg/kg of AT).
- Group V: The optimized formulation-treated group that received oral treatment with the optimized AT-NLCs (equivalent to 25 mg/kg of AT).

#### 2.11.3. Biochemical Assay 

After two weeks of treatment, blood samples were collected and centrifuged at 5000 rpm for 10 min to separate the plasma. The collected plasma samples were assayed for total cholesterol (TC), low-density lipoprotein (LDL), high-density lipoprotein (HDL), and triglyceride (TG) levels using a commercially available diagnostic kit (Span Diagnostic Ltd., Surat, India) [11].

#### 2.11.4. Histopathological Analysis

In the last part of the aforementioned experiment, the rats were scarified by decapitation under isoflurane anesthesia, and tissue specimens of rats’ liver from different groups were fixed in 10% neutral buffered formalin for 24 h. Successively, samples were washed with distilled water followed by dehydration in alcohol. Samples were cleared in xylene and fixed in paraffin beeswax blocks at 56 °C for 24 h. Sections (5 mm) from the paraffin blocks were sliced by a sledge microtome (3 μm thickness), deparaffinized, stained with hematoxylin/eosin, and examined by an independent pathologist. All samples were photomicrographed for steatosis (fatty change in the liver) and damage parameters of liver cells such as necrosis, pyknosis, and congestion using a binocular microscope (Leica, Wetzlar, Germany).

## 3. Results and Discussion

### 3.1. Preliminary Study

**Figure 1:**
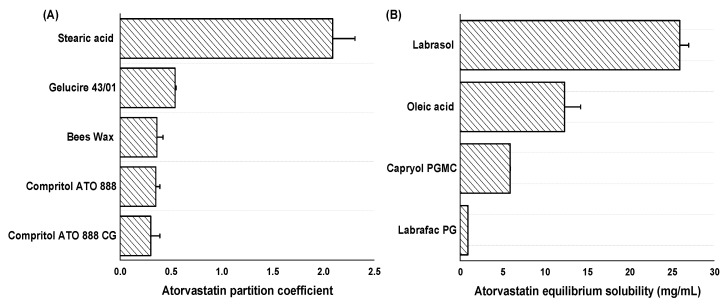
Results of the preliminary Atorvastatin partition coefficient and equilibrium solubility study: (**A**) Partition coefficient of Atorvastatin in different solid lipids; (**B**) The solubility of Atorvastatin in different types of oils.

The selection of solid and liquid lipids for the formulation of AT-NLCs has a significant influence on the drug entrapment efficiency. The drug should have a high affinity towards both solid and liquid lipids in order to ensure the highest possible drug loading and to prevent drug precipitation following dilution under physiological conditions [33]. A partition coefficient study was carried out for different solid lipids to select the most appropriate one that had the highest partition coefficient. The results are summarized in Figure 1A. The highest AT partition coefficient was observed for Stearic acid (2.09 ± 0.22) followed by Gelucire 43/01 (0.54 ± 0.01), Beeswax (0.36 ± 0.06), Compritol ATO 888 (0.35 ± 0.04), and Compritol ATO 888 CG (0.30 ± 0.09). Consequently, both Stearic acid and Gelucire 43/01 were selected as solid lipids for the experimental screening design.

The solubility of AT in different oils is shown in Figure 1B. The highest AT equilibrium solubility was observed in Labrasol (25.90 ± 1.05 mg/ mL) followed by Oleic acid (12.30 ± 1.89 mg/mL), Capryol PGMC (5.87 ± 0.02 mg/mL), and Labrafac PG (0.89 ± 0.01 mg/mL). Consequently, both Labrasol and Oleic acid were selected as liquid lipids for the experimental screening design.

### 3.2. Combined D-optimal Screening Design

The quality-by-design approach is gaining greater interest in developing and optimizing pharmaceutical formulations. Over many years using experimental designs, it is possible to rule out that the experimental design can be considered a double-edged sword as it outputs results whether the used factors or levels are carefully and correctly chosen or not or whether the used design fits the studied problem or not. Across the literature, various articles use the experimental design to optimize formulation parameters using designs that cover only a small fraction of the actual design space. It is obvious that they usually skip the step of screening the most important factors that really need to be optimized and at most they depend on knowledge-based trials or previously studied factors in similar instances. Developing a screening design for each drug formulation of specific criteria is essential to determine the important factors related to the working design space and to develop an optimization design for the factors that actually participate in the formation of an optimum formula with reliable characteristics. In this regard, formulations that are studied and optimized using the quality-by-design approach are considered more scientifically accredited and more reliably stable than the ones using one variable at a time approach or even using surged experimental designs. In an attempt to prepare an efficient screening experimental design with the most suitable factors influencing the preparation of AT-NLCs, a comprehensive screening design with different processes and mixture factors was developed to consider all aspects of the working design space. The 46 experiment D-optimal screening design with five replicates and five lack-of-fit experiments was blocked into five days to consider day to day variations and errors. The mixed factors were A, B, C, and D (Labrasol, Oleic acid, Gelucire 43/01, and Stearic acid, respectively), and the process factors were E, F, G, H, and J (surfactant concentration, homogenization speed, sonication time, total lipids, and the solid liquid ratio, respectively), which represented the numeric factors; K, L (lecithin incorporation and type of surfactant) represented categoric factors. A combined D-optimal design was chosen with two levels and eleven factors to help study this large number of factors with minimum cost and resources, allowing constraints for mixture-based factors and handling different levels for each one of the studied factors (Table 1). The experimental domain of the two-level D-optimal screening experimental design of the formulation parameters for the 46 runs and their measured responses are summarized in Table S1 of the Supplementary Materials. The observed response coefficients of the experimental runs along with the Analysis of Variance (ANOVA) results calculated for measured responses such as the *p*-value are summarized in Table 2 and Equations (3)–(5). Statistical analysis using ANOVA revealed that the sequential model suggested for evaluating the different parameters was linear.

ZP = −10.15 × AC − 6.31 × AD − 4.62 × BC − 5.24 × BD + 2.63 × ACF − 4.66 × ACJ + 6.17 × ACK + 3.74 × ADG + 5.08 × ADH + 2.18 × ADJ + 4.50 × ADK − 2.27 × BCE + 3.52 × BCF − 3.88 × BCG + 2.62 × BCH − 4.05 × BCJ − 2.98 × BCL + 2.60 × BDH(3)

PS = 14.44 × AC + 36.15 × AD + 5.23 × BC + 8.52 × BD + 6.40 × ACE − 8.25 × ACF + 3.76 × ACG + 6.50 × ACH − 5.92 × ACJ + 8.09 × ACK − 6.71 × ACL + 6.75 × ADE + 4.09 × ADF − 6.20 × ADG − 18.72 × ADJ − 12.35 × ADK(4)

PDI = −10.16 × AC − 0.24 × AD − 0.32 × BC − 0.22 × BD + 0.11 × ACF − 0.06 × ACH + 0.19 × ACJ − 0.06 × ACK + 0.08 × ACL + 0.06 × ADE + 0.09 × ADJ − 0.08 × ADK − 0.05 × BDK(5)

#### 3.2.1. Effect of the Independent Variables on the Zeta Potential (ZP)

**Figure 2:**
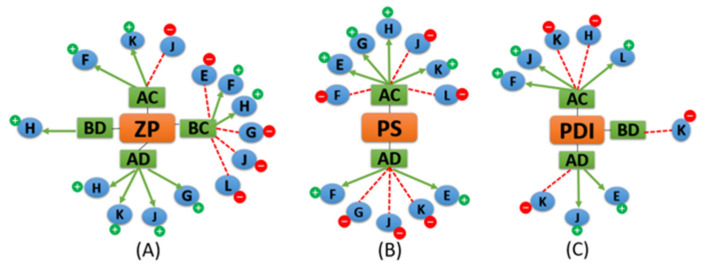
A summary diagram of the responses’ three-way interaction coefficients of the mixed variables (A: Labrasol, B: Oleic acid, C: Gelucire 43/01, D: Stearic acid) with different process variables (E: Surfactant concentration, F: Homogenization speed, G: Sonication time, H: Total lipid, J: Solid/Liquid lipid ratio, K: Lecithin, and L: Surfactant type) and their impact on (**A**) zeta potential (ZP); (**B**) average particle size (PS); and (**C**) polydispersity index (PDI).

**Figure 3:**
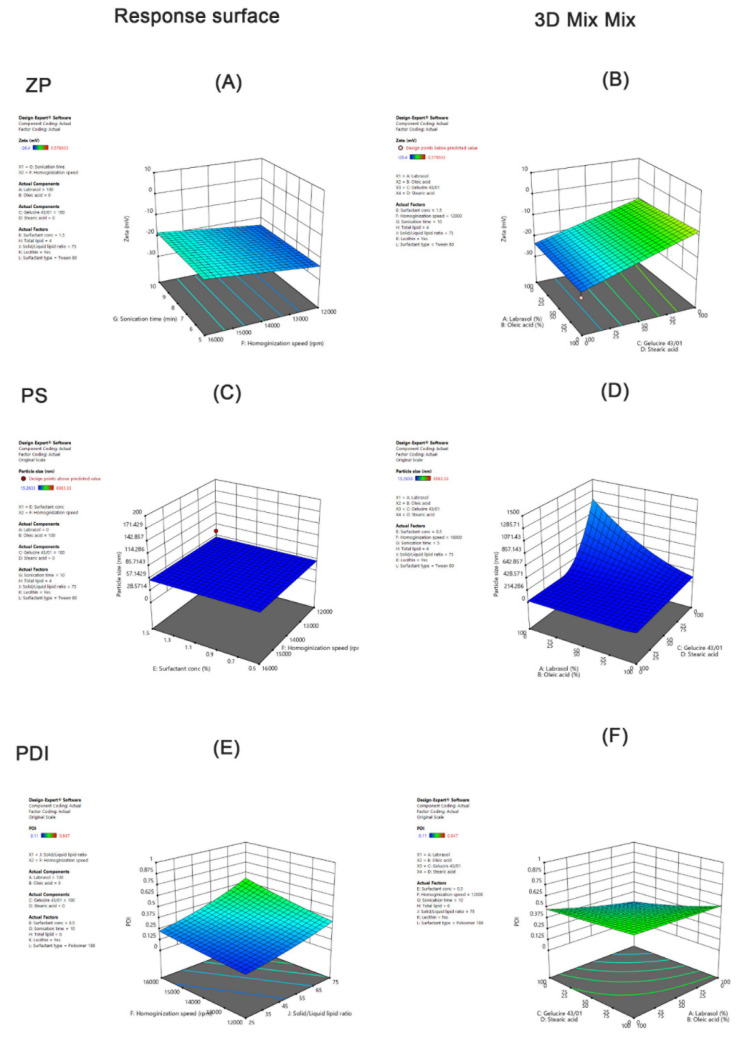
3D surface response plots showing the effects of the process factors on: (**A**) zeta potential; (**C**) particle size; and (**E**) PDI, and 3D MIX-MIX plots showing the effects of the mixed factors on: (**B**) zeta potential; (**D**) particle size; and (**F**) PDI.

The statistical interpretation of the design coefficients demonstrated the significance of the two-way interactions between the liquid lipid type and the solid lipid type in conjunction with other factors and their effect on different dependent variables. A positive sign indicated a synergistic effect on ZP (increase), while a negative sign indicated an antagonistic effect on ZP (decrease). Our objective was to maintain zeta potential values within the −35 to −25 mV range. Accordingly, interactions with negative values (antagonistic effect) were desirable. Careful inspection of the equation coefficients of ZP indicated that AC (combination of Labrasol and Gelucire 43/01), AD (combination of Labrasol and Stearic acid), BC (combination of Oleic acid and Gelucire 43/01), and BD (combination of Oleic acid and Stearic acid) were favorable and decreased ZP values (Equation (3)). Hence, it was important to relate mixed factor interactions with process factors to identify liquid and solid lipids that produced AT-NLCs with an optimum ZP value. For example, inspecting Equation (3) showed that ACK (Labrasol and Gelucire 43/01 with lecithin), ACF (Labrasol and Gelucire 43/01 with homogenization speed), ADH (Labrasol and Stearic acid with high total lipid), and ADJ (Labrasol and Stearic acid with solid/liquid lipid ratio) were unfavorable (Figure 2A). On the contrary, ACJ (Labrasol and Gelucire 43/01 with solid/liquid lipid ratio) was favorable (Figure 2A). The ANOVA results suggested that no significant influence of changing either the type of surfactant or its concentration on ZP. For confirmation of ANOVA results and greater exploration of the design space, the combined effect of (A, B, C, and D) presented (Figure 3A) showed that when Labrasol was the liquid lipid and Gelucire 43/01 was the solid lipid, ZP values were favorable (decreased) to about −23.7 mV. The negative charge was due to the anionic nature of the lipids [17].

Further, ZP of −35 to −25 mV is required for electrostatic stabilization for dispersed systems [34]. This result is attributed to the presence of Labrasol PEG chains that cover the particle surfaces that have a better effect on the zeta potential which is often the key factor in understanding how the dispersion and aggregation processes are applied [35]. As depicted (Figure 3B), decreasing the homogenization speed (F) and total lipid concentration (H) as well as increasing the percentage of the solid lipid ratio in the solid/liquid lipid mixture (J), and the sonication time (G) had favorable effects on ZP (decreased). The impact of the incorporation of lecithin in the formulation was also studied. Use of lecithin as a lipophilic emulsifier enhanced ZP values (decreased). This result agreed with Nnamani et al., who stated that the use of mixed hydrophilic and lipophilic surfactants gives better stability to the dispersed phase [36]. On the other hand, changing the concentration of the surfactants had no impact on ZP, possibly due to the nonionic nature of surfactants used (Tween 80 and Poloxamer 188) that have low or zero impact on the ZP [37].

#### 3.2.2. Effect of the Independent Variables on Particle Size (PS)

Lower values of PS are desirable for better formulated AT-NLCs. The PS of nano formulations is an important factor that affects the stability, bioavailability, and lymphatic transport of PWSDs [15,38]. Wu et al. reported that NLCs with smaller PS (50–100 nm) were able to enhance absorption of a poorly soluble drug (Repaglinide) compared to larger PSs [39]. The statistical interpretation of the design coefficients indicated that A (Labrasol) and C (Gelucire 43/01), AD (combination of Labrasol and Stearic acid), BC (combination of Oleic acid and Gelucire 43/01), and BD (combination of Oleic acid and Stearic acid) had synergistic effects on PS (increased) (Table 2, Equation (4)). Surfactant type, solid/liquid lipid ratio, and homogenization speed had antagonistic effects on PS (decreased) when combined with AC, while the remaining factors combined with AC had significant synergistic influences on PS. Sonication time, type of surfactant, and total lipid concentration had no significant influence on PS (Figure 2B). For confirmation of ANOVA results and greater exploration of the design space, Figure 3C shows that the increase in Oleic acid and Gelucire 43/01 resulted in enhanced (decreased) PS as low as 53.7 nm. For instance, Eleraky et al. [40] reported that using different liquid lipid types (Oleic acid, Labrasol, Capryol 90, and Miglyol 840) for the preparation of Temazepam-NLCs led to variations in the final particle size, where a smaller particle size was produced when Oleic acid was used while Labrasol produced a larger particle size. This may be due to the higher HLB of Labrasol compared to Oleic acid (12 and 1, respectively). Gelucire 43/01 also contains mono- and diglycerides in addition to polyethylene glycol esters of fatty acids that impart certain surface-active properties that can be the reason for the reduced PS [41]. The high melting point of stearic acid in comparison to Gelucire 43/01 (69.5 °C and 43 °C, respectively) led to higher melt viscosity and consequently decreased the efficiency of the homogenization step in reducing the particle size [17]. As depicted (Figure 3D), decreasing the homogenization speed and increasing the sonication time led to a smaller PS of AT-NLCs, in agreement with Chaudhary et al. [42]. Furthermore, increasing the surfactant concentration led to a large PS (Figure 3D). A similar finding was observed by Salami et al. A larger PS was developed when elevated concentrations of the surfactant were used in the aqueous phase, causing a higher viscosity [43]. Likewise, increasing the total lipid concentration as well as decreasing the percentage of solid lipids in the solid/liquid lipid mixture led to a large PS. The same results were reported by Chaudhary et al.: prepared Silymarin-NLCs had different solid to liquid ratios and increasing the solid lipid ratio led to increased PS [42]. Incorporation of lecithin led to a smaller particle size of the developed formulation. Emami et al also reported that the absence of Lecithin in the oil phase of NLCs possibly caused an increase in PS due to the increase in the micelle core viscosity [44].

#### 3.2.3. Effect of the Independent Variables on PDI

PDI measures the PS distribution in the formulation and ranges from 0 to 1. Low PDI values (0.1 to 0.25) display a fairly narrow distribution of size and encourage long-term nano dispersion stability, whereas values greater than 0.5 suggest a very large size distribution [45]. Lower values of PDI are desirable for a lower variation in the AT-NLC formulation. The statistical interpretation of the design coefficients indicated that Labrasol and AC (Gelucire 43/01), AD (combination of Labrasol and Stearic acid), BC (combination of Oleic acid and Gelucire 43/01), and BD (combination of Oleic acid and Stearic acid) decreased PDI (Table 2, Equation (5)), which is again favorable. In contrast to PS, the combined interaction of the solid/liquid lipid ratio and surfactant type with AC increased PDI, while the total lipid concentration and incorporation of lecithin decreased PDI. **(**Figure 2C). No significant effect of the sonication time and total lipid concentration on the PDI was observed. For confirmation of ANOVA results and greater exploration of the design space, Figure 3E shows that using Labrasol as the liquid lipid and Gelucire 43/01 as the solid lipid resulted in enhanced (decreased) PDI values as low as 0.14. As illustrated from Figure 3F, PDI decreased upon decreasing the surfactant concentration and solid lipid ratio in the liquid lipid mixture. Furthermore, the incorporation of lecithin and use of Tween 80 decreased the PDI values (increased).

#### 3.2.4. Summary of the Screening Results

From the screening experimental design, we could conclude the factors that had significant effects on the parameters for AT-NLC preparation that need to be qualified for further optimization and the levels to be set for the insignificant factors. Among the mixed factors, a compromise was made, and Gelucire 43/01 (solid lipid) and Labrasol (liquid lipid) were used in the optimization step to enhance both ZP and PDI. Among the categorical process factors, lecithin and Tween 80 were important to enhance ZP and PS. Among the numerical process factors, center levels for total lipid concentration (5%) and sonication time (7.5 min) were used to enhance the AT-NLC formulation parameters. Three parameters of the AT-NLC formulation were further tested and selected as independent factors (the homogenization speed, solid/liquid ratio, and surfactant concentration) using an optimization design. The measured responses were the average particle size (PS), zeta potential (ZP), polydispersity index (PDI), and entrapment efficiency (EE%).

### 3.3. Central Composite Design (CCD)

A response surface methodology using a 3-factor, 5-level central composite design (CCD) was applied to determine the optimum levels of the formulation parameters of AT-NLCs, using a minimum number of experiments. A rotatable CCD was used with star points at α = 1.68 to generate information for the design space sphere from all directions. Then, 20 experiments were proposed in random order to test the major significant factors: the homogenization speed (A), solid/liquid lipid ratio solid lipid (B), and the concentration of the surfactant (C) (Table 3). PS, PDI, ZP, and EE% were selected as dependent optimizable response variables.

According to the CCD matrix generated by the software, the CCD method provides a potential benefit in experimental design by adding six replicated center points. The replication was frequent in order to improve the precision of the experiment. The observed response coefficients of the experimental runs along with ANOVA results calculated for measured responses such as the *p*-value are summarized in Table 4 and Equations (6)–(9).

PS = 57.22 + 32.05 × A − 16.87 × B + 15.47 × AC − 12.86 × BC + 18.38 × AA^2^ + 31.10 × BA^2^ + 33.62 × AA^2^B − 32.17 × AA^2^C − 47.37 × ABA^2^ (6)

PDI = 0.54 + 0.11 × AC + 0.06 × AA^2^ − 0.05 × BA^2^ − 0.09 × ABC (7)

ZP = −26.25 − 2.42 × B + 1.22 × C + 4.14 × AB − 1.56 × AC − 1.77 × AA^2^ (8)

EE = 82.71 − 5.40 × B + 2.35 × CA^2^ − 4.39 × AA^2^C (9)

Statistical polynomial analysis using ANOVA revealed that the sequential model suggested for evaluating the particle size, PDI, and EE% was quadric except that of ZP was cubic. Moreover, the insignificant terms (with *p*-value < 0.05) were removed using a backward elimination procedure. The models for each dependent variable were checked for fitting, and both R^2^ and adj. R^2^ values were in the range of 0.84–0.99 with a difference less than 0.2 indicating that both R^2^ and adj. R^2^ were interrelated. In addition, all the models showed a non-significant lack-of-fit, which again showed good fitting.

#### 3.3.1. Effect of the Independent Variables on Particle Size (PS)

**Figure 4:**
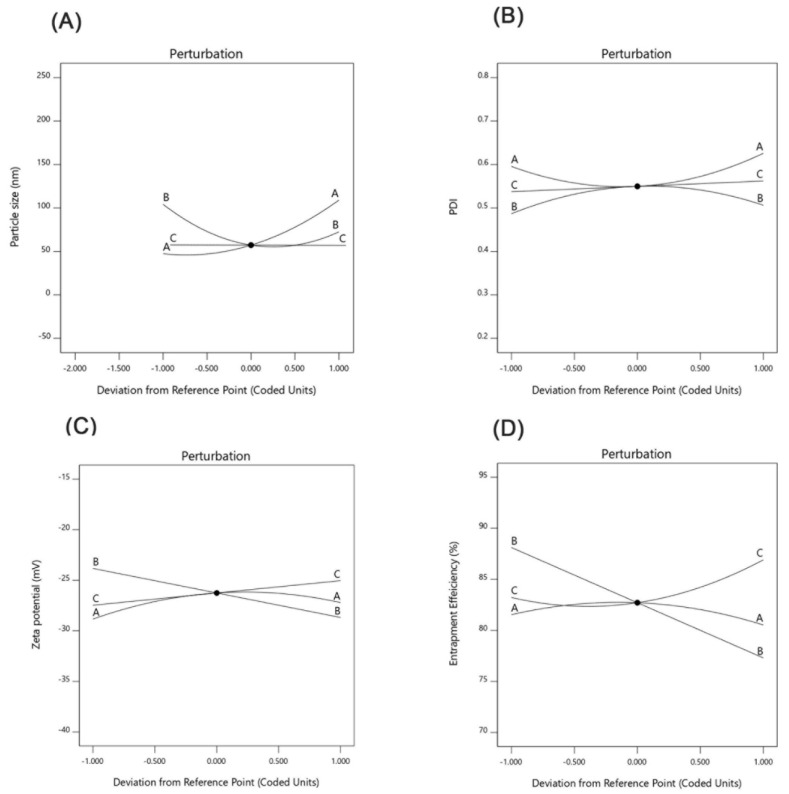
Perturbation plots showing the effect of each independent variable alone on the (**A**) particle size (PS), (**B**) polydispersity index (PDI), (**C**) zeta potential (ZP), and (**D**) entrapment efficiency (EE%).

Inspection of the coefficients of the ANOVA results in Table 4 showed that homogenization speed (A) had a prominent synergistic effect on particle size, while the solid lipid ratio in the solid/liquid lipid mixture had a lesser effect and antagonistic influence on particle size. The surfactant concentration had no significant influence on the PS. Quadratic interaction and interaction with quadratic terms contributed distinctly to the PS of the generated AT-NLC formulation. The interaction effect of AC (homogenization speed and surfactant concentration) influenced PS synergistically, while the interaction effect of BC (solid/liquid ratio and surfactant concentration) had an antagonistic influence on PS. Perturbation plots were presented to show the effect of each factor on a specific response when all other factors were held constant at a reference point (Figure 4A). Curvature or slope steepness indicates sensitiveness to a specific factor. Again, it was clear from the plot steepness that factor A had a more prominent effect on PS than factor B. In addition, the plot direction confirmed that factor A had a synergistic effect on PS while factor B had an antagonistic effect on PS. 

**Figure 5:**
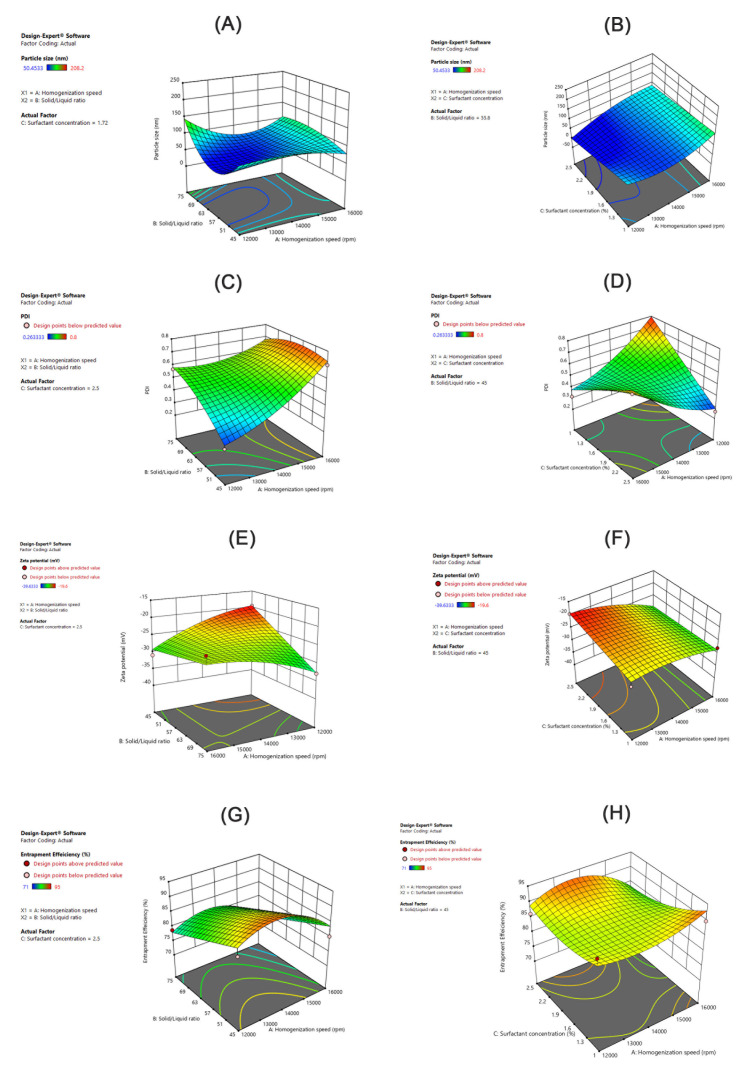
3D surface response plots showing interaction effects of independent variables on PS, PDI, ZP, and EE%.

For confirmation of ANOVA results and greater exploration of the design space, the response surface for the interaction effect of homogenization speed and the solid lipid ratio in the solid/liquid lipid mixture on particle size was explored (Figure 5A). Figure 5A shows that decreased PS values were obtained using a medium homogenization speed and medium solid lipid ratio for the solid/liquid lipid mixture values. This agreed with Chaudhary et al. [42], who prepared Silymarin-NLCs with different homogenization speeds and reported that either low speed or high speed was not enough to produce a small particle size but an intermediate homogenization speed provided outstanding size reduction. This may be attributed to the fact that at low to medium velocity, particle size decreases due to a breakdown in particles, but drastic alteration in particle size was seen at higher velocity due to excessive collisions between particles which formed aggregates that finally increased PS [42]. 

On the other hand, the incorporation of a high solid lipid (Gelucire 43/01) ratio in the solid/liquid lipid mixture means the incorporation of more mono- and diglycerides besides polyethylene glycol esters of fatty acids that may impart certain surface-active properties. Accordingly, PS could be reduced [41]. The reduction in PS was to a certain extent (around 60%) due to the solid/liquid lipid ratio, and then an increase in PS was observed because of the reduction in the liquid lipid availability.

Upon the increase in the liquid to solid lipid ratio, the surface tension of the melted lipid drops can be easily reduced by the surfactants used, which help the breakdown of the lipid particles to a smaller PS [46]. It was also observed that increasing the surfactant concentration led to a further decrease in PS (Figure 5B). This could be explained based on the fact that high surfactant concentrations reduce the interfacial tension. Accordingly, better lipid partitioning during emulsification leads to a smaller PS [47].

#### 3.3.2. Effect of the Independent Variables on PDI 

As illustrated in Table 4, the studied variables had no prominent effect on the polydispersity index (PDI). Slight effects were observed for the interaction term of AC and the quadratic terms of A^2^ and B^2^. These findings agreed with the inspection of the PDI perturbation plot (Figure 4B). Exploration of response surfaces (Figure 5C,D) was more useful as they showed the effect of factor interactions on the PDI. Values between 0.1–0.25 of PDI were obtained by decreasing both the homogenization speed and solid lipid ratio in the lipid mixture and increasing the surfactant concentration. 

#### 3.3.3. Effect of the Independent Variables on the Zeta Potential (ZP)

As illustrated in Table 4, the surfactant concentration and interaction of AB had a synergistic effect on ZP while the solid lipid ratio in the solid/liquid lipid mixture and interaction of AC had an antagonistic influence on ZP. The homogenization speed (A) had no significant effect on the ZP. All these effects were not prominent as seen in the corresponding perturbation plot (Figure 4C). Exploration of response surfaces of interaction effects (Figure 5E,F) showed that ZP values in the range of −35 to −25 mV were obtained by decreasing both the homogenization speed and solid lipid ratio in the solid/liquid lipid mixture and increasing the surfactant concentration (as previously proposed for PDI). It was found that the ratio of Gelucire 43/01 to Labrasol had a positive influence in the ZP. Different findings were mentioned by L. Kiss et al [48], who found that as the solid lipid ratio increased, there was a negative influence on ZP. Further, it was observed that the increase in the Tween 80 concentration had a negative effect on the ZP of AT-NLCs. These results suggest that Tween 80 was adsorbed on the particle surfaces due to its higher surface activity by displacing the endogenous surface-active compounds [49]. Since Tween 80 is a non-ionic surfactant, the increase in the concentration of Tween 80 adsorbed on the particulate surface resulted in a decrease in the magnitude of the surface net charge. These results are consistent with previous work, e.g., of Witayaudom and Klinkesorn [50], who prepared NLCs of Rambutan (*Nephelium lappaceum* L.) kernel fat which stabilized with different concentrations of Tween 80. Unlike particle size, however, the effect of the homogenization speed on ZP could not be fitted to this model.

#### 3.3.4. Effect of the Independent Variables on the Entrapment Efficiency (EE%)

As illustrated in Table 4, the solid lipid ratio in lipid mixture had an antagonist effect on the EE%. The same finding could be observed by the inspection of perturbation plots (Figure 4D), where we could conclude that a high EE% could be achieved by decreasing factor B. Exploration of response surfaces of interaction effects (Figure 5G,H), showed that EE% higher than 90% could be achieved by decreasing factor B and either increasing factor A and decreasing factor C or using median factor A and increasing factor C. This may be attributed to the higher availability of liquid lipids at the expense of solid lipids that led to increases in holding the drug in the NLC amorphous matrix as previously reported by Song et al. [46], who prepared Flurbiprofen-loaded solid lipid nanoparticles (SLN) and nanostructured lipid carriers (NLCs) with the aim to clarify the superiority of NLCs over SLN due to the presence of liquid lipids.

#### 3.3.5. Selection of the Optimized AT-NLC Formulation

**Figure 6:**
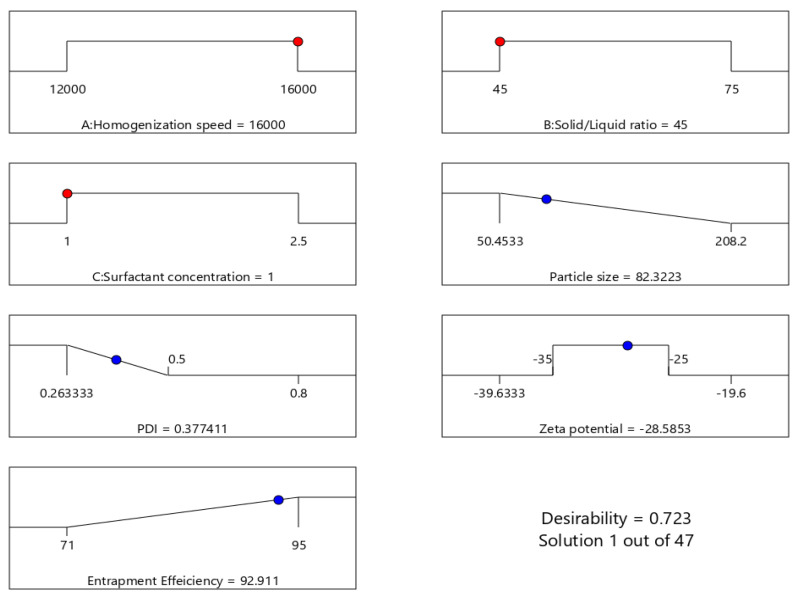
Optimization ramps for the studied independent variables with the predicted value of each measured formulation parameter.

According to the developed polynomial equations (Equations (6)–(9)), optimization was done using the desirability approach, in order to obtain the levels of A, B, and C that will minimize PZ and PDI in the range 0.1–0.25 and ZP in the range −35 to −25 mV, while maximizing EE%. The desirability value varies from (0–1). A formulation is judged acceptable when the desirability value approaches 1. Therefore, the formulation that had the maximum possible desirability value was selected. The optimum criteria for AT-NLC formulation were a homogenization speed of 16000 rpm, solid lipid ratio in the solid/liquid lipid mixture of 45%, and surfactant concentration of 1% with a desirability of 0.723 (Figure 6). To validate the values of the responses, the obtained results from the optimized formulation were compared to those predicted by the software. The experimental values for the response of the optimal formula were consistent with the expected values provided by the software, confirming the validity of the design summarized in Table 5.

### 3.4. Transmission Electron Microscopy (TEM)

**Figure 7:**
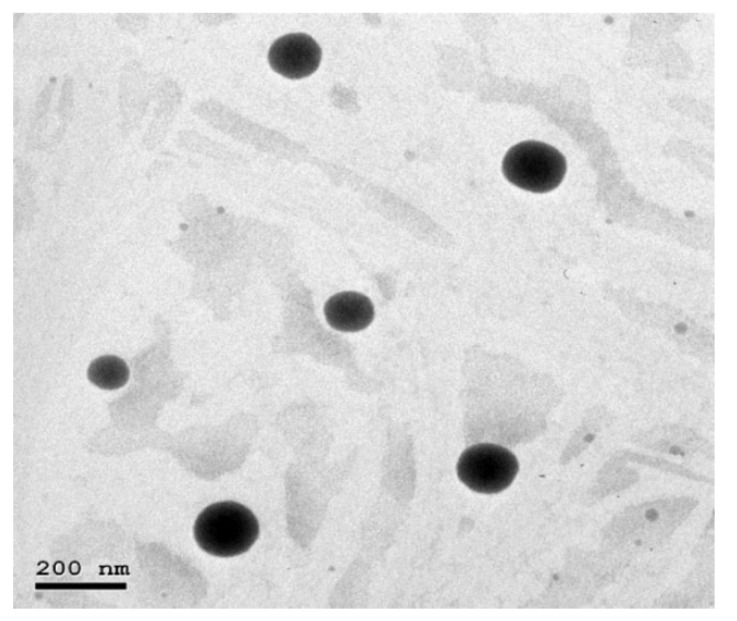
TEM image of the optimized AT-NLC formulation.

TEM was performed to study the shape and size distribution of the optimized AT-NLCs. As shown in Figure 7, the TEM image of the optimized AT-NLC formulation revealed that the prepared AT-NLCs possessed a uniform spherical shape. The noticeable difference between PS measurement by the two methods (TEM vs. dynamic laser scattering) can be explained by the difference in the measuring techniques of both methods. TEM measurement is a number-based PS measuring technique whereas dynamic laser scattering is an intensity-based PS measuring method.

### 3.5. Differential Scanning Calorimetry (DSC)

**Figure 8:**
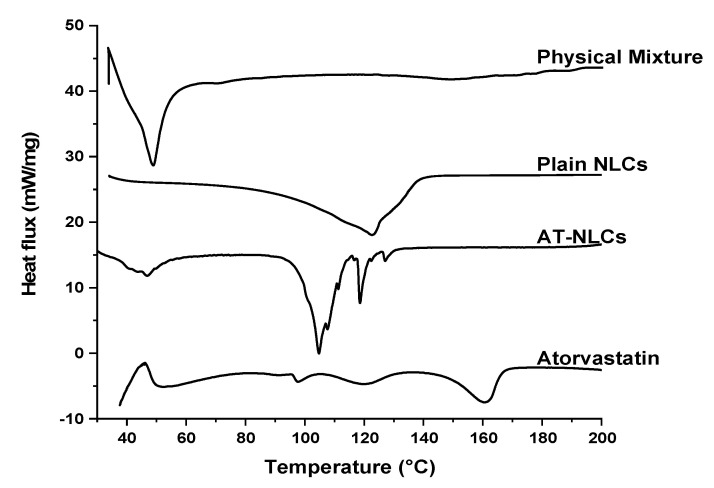
DSC thermograms of Atorvastatin, optimized AT-NLC formulation, plain NLC formulation, and the physical lipid mixture.

DSC is a useful tool to study the thermal behavior of both drug(s) and excipients as well as their possible interactions within the drug formulations. For instance, DSC thermograms can be used to characterize the melting and crystallization behavior of crystalline solid lipids (Gelucire 43/01) as well as the pure drug (AT). DSC thermograms of pure AT drug powders showed two small broad endothermic peaks at 97.7 °C and 120.5 °C (Figure 8) that represent the water loss from pure AT powders. AT powders have reported to have about 4–6% *w*/*w* of both loosely and tightly bound water [51]. Additionally, the DSC thermogram of AT showed a melting endothermic peak at 160.8 °C. Gelucire 43/01 showed a melting endothermic peak at 48.9 °C in the physical mixture. However, there was a shift in the Gelucire 43/01 melting endothermic peak to more than 100 °C in both the lyophilized plain and loaded optimized NLC formulation. This may be attributed to the reduction in PS to the nanometer range (83.80 ± 1.13 nm) of the optimized NLC formulation and the presence of Tween 80 and Lecithin as surfactants. The same observation was reported by Elmowafy et al. [17]. The lyophilized AT-NLC formulation did not show the characteristic melting endothermic peak of AT at 160.8 °C, suggesting the presence of AT in the amorphous form within the optimized AT-NLC formulation. The conversion of AT from the ordered crystalline form to the highly energetic disordered amorphous or molecularly dispersed form within the optimized AT-NLC formulation is beneficial for enhancing the AT dissolution rate and hence bioavailability [52].

### 3.6. Fourier-Transform Infrared Spectroscopy (FTIR)

**Figure 9:**
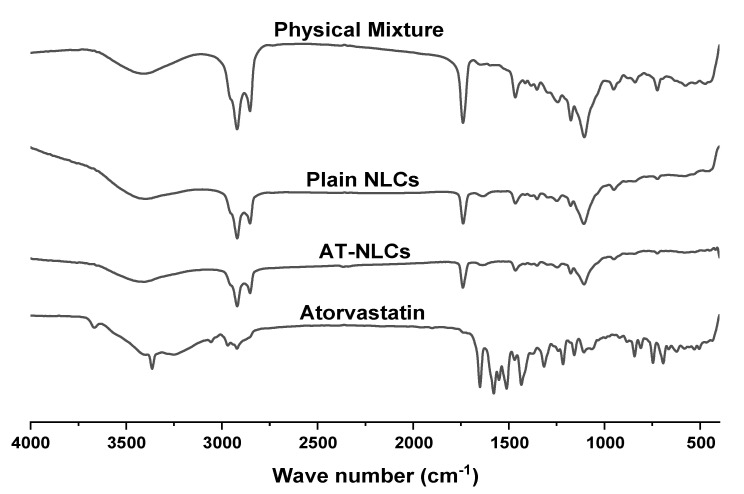
FTIR spectra of Atorvastatin, optimized AT-NLC formulation, plain NLC formulation, and the physical lipid mixture.

FTIR spectra are useful to study the interactions between drugs and lipid excipients. The appearance of a new peak or a shift in the spectral prominent peaks as well as the broadening or vanishing of an existing peak may indicate a molecular interaction. FTIR spectra of Atorvastatin, the optimized AT-NLC formulation, plain NLC formulation, and physical lipid mixture are shown in Figure 9. The FTIR spectrum of pure AT showed distinctive peaks at 3668.9 cm^−1^ (non-hydrogen-bonded O-H), 3364.2 cm^−1^ (N-H stretching), 3250.4 cm^−1^ (symmetrical O-H stretching), 2968.87 cm^−1^ (C-H stretching), 2920.66 cm^−1^ (C-H, aromatic), 1650.77 cm^−1^ (asymmetric C=O stretching), 1578.45 cm^−1^ (N-H bending), 1511.92 cm^−1^ (C-N stretching), 1434.78 cm^−1^ (O-H bending), and 1380.78 cm^−1^ (C-O stretching) of the carboxyl group, which were already reported in many studies [11,53]. AT-NLC spectrum revealed the disappearance of the 3668.9 cm^−1^ (non-hydrogen-bonded O-H) and 3364.2 cm^−1^ (O-H stretching) AT peaks compared to the pure AT spectrum. This may suggest Hydrogen bonding interactions of both AT and lipids at the molecular level.

### 3.7. In Vitro Release Studies

**Figure 10:**
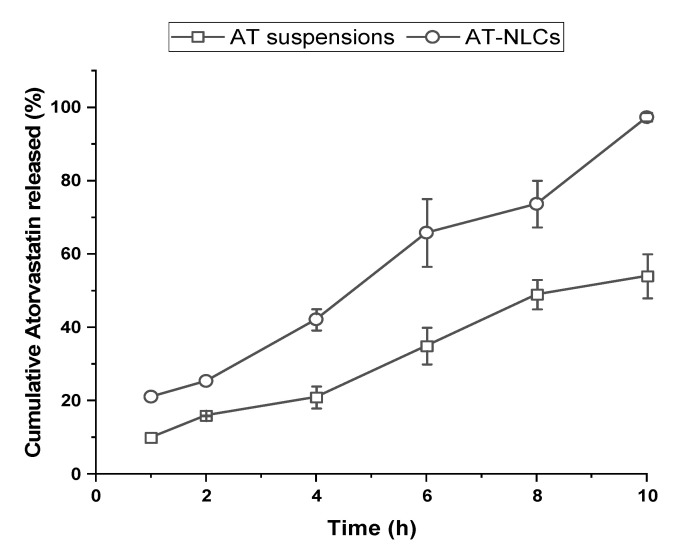
In vitro release profiles of AT suspensions and the optimized AT-NLC formulation in phosphate buffer, pH 6.8.

The cumulative AT in vitro release profiles from both AT suspensions and the optimized AT-NLC formulation in phosphate buffer, pH 6.8, are shown in Figure 10. A marked enhancement of AT in vitro release from the optimized AT-NLC formulation was observed compared to the drug suspensions. Complete AT release from the optimized formulation was observed within 10 h while only 60% was released from the AT suspensions within the same time period. Additionally, no burst AT release was noticed from the optimized AT-NLC formulation. This may be attributed to the presence of the PEG moiety of Labrasol at the surface of the NLCs causing a barrier for AT diffusion [35]. The relatively elevated cumulative release rate of AT-NLCs was presumably due to the disordered amorphous state of the AT that exhibited a higher dissolution rate and aqueous solubility [54].

In order to study the AT release kinetics from the optimized AT-NLC formulation, the release profile data were fitted to various release models. The selection of the best fit was based on the highest correlation coefficient (R^2^) value. The AT release profile from the optimized formulation in phosphate buffer, pH 6.8, was best fitted by the Higuchi diffusion model (R^2^ = 0.9665). The fitting R^2^ values of the other kinetics release models were in the following order: Krosmear Peppas (R^2^ = 0.9656); zero-order kinetics (R^2^ = 0.94); Hixso–Crowell model (R^2^ = 0.9027); and first-order kinetics (R^2^ = 0.878).

### 3.8. In Vivo Pharmacodynamic Study

#### 3.8.1. Biochemical Evaluation of Rats

After four weeks of high-fat diet administration, there was a significant increase (*p*-value < 0.0001) in total cholesterol (TC), triglycerides (TG), and low-density lipoprotein (LDL) serum levels. On the other hand, high-density lipoprotein (HDL) serum levels were decreased in Group II, suggesting successful induction of hyperlipidemia compared to Group I. Group III, group IV, and group V were treated for two successive weeks with AT suspensions, Lipitor^®^ 20, and the optimized AT-NLCs formulation, respectively. The three groups showed a significant TC level decrease (*p*-value < 0.0001) compared to the positive control group (Group II). The serum TC, TG, LDL, and HDL levels of the five groups and their ANOVA results are summarized in Table 6.

All treatment groups (Group III, IV, and V) showed a significant enhancement of the experimental animals’ TC, TG, and LDL profile compared to the non-treated positive control group (Group II) and a non-significant serum HDL level enhancement. Compared to Group II, the serum levels of experimental animals treated with the optimized AT-NLC formulation showed a significant decrease (*p*-value < 0.0001) in TC levels (~2.2-fold), TG levels (~1.5-fold), and LDL levels (~6.4-fold). On the other hand, a non-significant increase in serum HDL levels (~1.5-fold) was observed. Additionally, Group V, treated with the optimized AT-NLC formulation, showed a marked decrease (~1.5-fold) in serum TC levels compared to the other treatment groups with either AT suspensions or the Lipitor^®^ 20 commercial preparation.

#### 3.8.2. Histopathological Evaluation

The Batts–Ludwig system is the most extensively used scale for assessing both the grade (amount of necroinflammatory activity) and the stage (degree of fibrosis) of chronic hepatitis [55]. A summary of the Batts–Ludwig grading and stage of the five experimental animal groups is illustrated in Table 7.

A better score was observed for the AT suspensions as well as the optimized AT-NLC formulation, suggesting a good response to the treatment regimen. Microscopic photos representing both the inflammatory changes and fatty changes of the experimental animals’ liver samples of the five groups are shown in Figure S1 of the supplementary data. The liver architecture of Group I (the negative control) was intact (Figure S1(Aa,c)). The histopathological photo showed plates of hepatocytes radiating from the central vein with uniform portal tracts. On the other hand, Group II (Figure S1(Ab,d))—the positive control—showed interface hepatitis, confluent necrosis, and lytic necrosis. The same findings were observed by Kassem et al. [8]. Additionally, Group II showed portal inflammation with moderate portal tract expansion as well as lymphocytic infiltration with mild spillage of lymphocytes into the limiting plate hepatocytes. Moreover, most hepatocytes showed the accumulation of triglycerides (steatosis) (Figure S1(Ba)). Group III, which was treated with AT suspensions, showed a mild expansion of a few portal tracts, suggesting portal inflammation, with lymphocytic infiltrated with a congested vessel as well as mild improvement of the hepatocyte structure. Additionally, there was a fatty change in the liver, as observed in Figure S1(Ae,g). Group IV, which was treated with Lipitor^®^ 20 mg, showed a mild expansion of a few portal tracts with lymphocytic infiltrate and congested vessels. Moreover, moderate portal tract inflammation, mild interface hepatitis, and a single focus of focal inflammation was observed (Figure S1(Af,h)). This may suggest mild recovery of the liver tissues compared to Group II. Group V, which was treated with the optimized AT-NLC formulation, showed evidence of recovery. It was observed that liver architecture was well-preserved. Additionally, plates of hepatocytes radiating from the central vein with uniform portal tracts and patent sinusoids were observed (Figure S1(Ak,i)). This suggests successful treatment with the optimized AT-NLC formulation. To sum up, the optimized AT-NLC formulation greatly enhanced the hepatic cell viability compared to the positive control group. The results of the histological studies are in good agreement with the serum lipid profiles of different groups, confirming that AT-NLC is superior to other groups.

## 4. Conclusions

In this study, we effectively prepared an optimized AT-NLC formulation by emulsification, applying high-speed homogenization, and subsequently by ultrasonication. Preliminary screening was successfully performed using a combined D-optimal experimental design. Based on all data from the formulation design, it is possible to conclude that among the mixed factors, a compromise should be made between Gelucire 43/01 as a solid lipid and Labrasol as a liquid lipid. Among categoric process factors, Lecithin and Tween 80 were important to enhance the ZP and PS values. Among numerical process factors, median levels for the total lipid concentration (5%) and sonication time (7.5 min) were used to enhance the NLC formulation properties. CCD optimization revealed that the optimum process variables for AT-NLC formulation were homogenization speed of 16,000 rpm, solid lipid ratio in the solid/liquid lipid mixture of 45%, and surfactant concentration of 1% with a desirability of 0.723. The optimized AT-NLCs demonstrated nanometric size (83.80 ± 1.13 nm), PDI (0.38 ± 0.02), surface charge (−29.65 ± 0.65 mV), and high drug incorporation (93.1 ± 0.04%). FTIR analysis showed no chemical interaction between Atorvastatin and the lipid mixture. DSC analysis of the AT-NLCs suggested the transformation of Atorvastatin crystal into an amorphous state. Administration of the optimized AT-NLCs led to a significant reduction (*p* < 0.001) in the rat serum levels of total cholesterol, triglycerides, and low-density lipoproteins. This change was histologically validated by reducing the relevant steatosis of the liver.

## Figures and Tables

**Table 1:** Build information for the combined D-optimal screening design.

Factor	Name	Unit	Type	Min	Max
A	Labrasol	%	Mixture 1	0	100
B	Oleic acid	%	Mixture 1	0	100
C	Gelucire 43/01	%	Mixture 2	0	100
D	Stearic acid	%	Mixture 2	0	100
E	Surfactant concentration	%	Numeric	0.5	1.5
F	Homogenization speed	rpm	Numeric	12,000	16,000
G	Sonication time	min	Numeric	5	10
H	Total lipid	%	Numeric	4	6
J	Solid/Liquid lipid ratio		Numeric	25	75
K	Lecithin		Categoric	Yes	No
L	Surfactant type		Categoric	Poloxamer 188	Tween 80

**Table 2:** Coefficients and ANOVA results for the effects of the studied combined D-optimal screening design factor combinations.

**Coefficients**	**AC**	**AD**	**BC**	**BD**	**ACE**	**ACF**	**ACG**	**ACH**	**ACJ**	**ACK**	**ACL**	**ADE**	**BDK**
ZP	−10.15	−6.31	−4.62	−5.24		2.63			−4.66	6.17			
*p*	<0.0001	<0.0001	<0.0001	<0.0001		0.0080			<0.0001	<0.0001			
PS	14.44	36.15	5.23	8.52	6.40	−8.25	3.76	6.50	−5.92	8.09	−6.71	6.75	
*p*	<0.0001	<0.0001	0.0018	<0.0001	0.0009	<0.0001	0.050	0.0009	0.0021	<0.0001	0.0007	0.0001	
PDI	−10.16	−0.24	−0.32	−0.22		0.11		−0.06	0.19	−0.06	0.08	0.06	−0.05
*p*	−0.4844	<0.0001	<0.0001	<0.0001		0.0004		0.0303	<0.0001	0.0229	0.0055	0.0113	0.0441
**Coefficients**	**ADF**	**ADG**	**ADH**	**ADJ**	**ADK**	**BCE**	**BCF**	**BCG**	**BCH**	**BCJ**	**BCL**	**BDH**	
ZP		3.74	5.08	2.18	4.50	−2.27	3.52	−3.88	2.62	−4.05	−2.98	2.60	
*p*		<0.0001	<0.0001	0.0159	<0.0001	0.0081	0.0004	<0.0001	0.0041	<0.0001	0.0009	0.0063	
PS	4.09	−6.20		−18.72	−12.35								
*p*	0.0125	0.0004		<0.0001	<0.0001								
PDI				0.09	−0.08								
*p*				0.0008	0.0023								

Note: A: Labrasol, B: Oleic acid, C: Gelucire 43/01, D: Stearic acid, E: Surfactant concentration, F: Homogenization speed, G: Sonication time, H: Total lipid, J: Solid/Liquid lipid ratio, K: Lecithin and L: Surfactant type; ZP: zeta potential, PS: particle size, and PDI: polydispersity index. The factor combination was considered insignificant when *p* > 0.05 and was removed from the table.

**Table 3:** Experimental runs and the observed responses for the central composite design.

Std	Run	A (%)	B (%)	C (%)	PS (nm)	PDI	ZP (mV)	EE (%)
8	1	16,000.00	75.00	2.50	79.79 ± 1.17	0.67 ± 0.01	−23.57 ± 0.72	71.00 ± 0.29
7	2	12,000.00	75.00	2.50	79.52 ± 4.64	0.57 ± 0.15	−32.87 ± 1.40	78.80 ± 0.25
17	3	14,000.00	60.00	1.75	56.92 ± 1.05	0.51 ± 0.01	−26.40 ± 0.72	86.80 ± 0.40
12	4	14,000.00	85.23	1.75	114.77 ± 6.41	0.45 ± 0.02	−31.53 ± 0.47	72.40 ± 0.40
16	5	14,000.00	60.00	1.75	54.00 ± 1.60	0.50 ± 0.13	−25.63 ± 1.81	85.30 ± 0.30
6	6	16,000.00	45.00	2.50	72.05 ± 2.52	0.70 ± 0.03	−30.97 ± 0.64	80.23 ± 0.25
20	7	14,000.00	60.00	1.75	67.95 ± 9.58	0.60 ± 0.14	−22.97 ± 1.23	83.30 ± 0.25
11	8	14,000.00	34.77	1.75	171.53 ± 4.57	0.45 ± 0.04	−23.20 ± 0.30	90.50 ± 0.45
4	9	16,000.00	75.00	1.00	132.17± 0.24	0.46 ± 0.04	−28.17 ± 0.64	80.00 ± 0.32
13	10	14,000.00	60.00	0.49	57.10 ± 5.40	0.59 ± 0.13	−27.17 ± 1.42	88.80 ± 0.10
18	11	14,000.00	60.00	1.75	55.43 ± 3.68	0.71 ± 0.06	−27.00 ± 1.61	83.00 ± 0.17
1	12	12,000.00	45.00	1.00	134.53 ± 14.97	0.73 ± 0.15	−26.77 ± 0.84	92.00 ± 0.10
2	13	16,000.00	45.00	1.00	87.37 ± 0.78	0.32 ± 0.01	−27.80 ± 1.63	89.70 ± 0.25
14	14	14,000.00	60.00	3.01	56.35 ± 2.23	0.55 ± 0.02	−26.43 ± 0.70	95.00 ± 0.30
9	15	10,636.41	60.00	1.75	53.28 ± 1.90	0.75 ± 0.02	−31.17 ± 0.84	79.27 ± 0.37
15	16	14,000.00	60.00	1.75	50.45 ± 1.26	0.52 ± 0.01	−24.67 ± 0.50	82.68 ± 0.12
19	17	14,000.00	60.00	1.75	56.22 ± 0.99	0.50 ± 0.01	−24.20 ± 0.62	81.90 ± 0.21
10	18	17,363.59	60.00	1.75	161.10 ± 11.91	0.80 ± 0.08	−29.53 ± 1.54	81.70 ± 0.15
3	19	12,000.00	75.00	1.00	208.20 ± 1.92	0.46 ± 0.02	−39.63 ± 0.81	74.90 ± 0.15
5	20	12,000.00	45.00	2.50	71.72 ± 0.06	0.26 ± 0.01	−19.60 ± 0.58	86.20 ± 0.25

Note: A: Homogenization speed, B: Solid/Liquid ratio and C: Surfactant concentration; ZP: zeta potential, Ps: particle size, and PDI: polydispersity index.

**Table 4:** Coefficient table for the effect of independent variables on dependent variables.

Parameters	Intercept	A	B	C	AB	AC	BC	AA^2^	BA^2^	CA^2^	ABC	AA^2^B	AA^2^C	ABA^2^
PS	57.22	32.05	−16.87			15.47	−12.86	18.38	31.10			33.62	−32.17	−47.37
p		<0.0001	0.0002			0.0001	0.0004	<0.0001	<0.0001			<0.0001	<0.0001	<0.0001
PDI	0.54					0.11		0.06	−0.05		−0.09			
p						0.002		0.0161	0.0300		0.0075			
ZP	−26.25		−2.42	1.22	4.14	−1.56		−1.77						
p			0.0006	0.04	<0.0001	0.046		0.0049						
EE	82.71		−5.40							2.35			−4.39	
p			<0.0001							0.0150			0.0269	

Abbreviations: A: Homogenization speed, B: Solid/Liquid ratio, C: Surfactant concentration; ZP: zeta potential, PS: particle size, and PDI: polydispersity index. The factor combination was considered insignificant when *p* > 0.05 and was removed from the table.

**Table 5:** Comparison between the predicted values and the actual values of the optimized AT-NLC formulation.

**Independent Variables**			**Dependent Variables**							
			**Predicted Value**				**Observed Value**			
**A**	**B**	**C**	**R1**	**R2**	**R3**	**R4**	**R1**	**R2**	**R3**	**R4**
rpm	%	%	nm		mV	%	nm		mV	%
16,000	45	1	82.32	0.38	−28.59	92.91	83.80 ± 1.13	0.38 ± 0.02	−29.65 ± 0.65	93.1 ± 0.04

Note: A: Homogenization speed, B: Solid/Liquid ratio and C: Surfactant concentration; R1: Particle size and R2: Polydispersity index, R3: Zeta potential, and R4: Entrapment efficiency.

**Table 6:** Serum total cholesterol (TC), triglycerides (TG), low-density lipoprotein (LDL), and high-density lipoprotein (HDL) levels of the five experimental animal groups.

Groups	TC	TG	LDL	HDL
Group I	80 ± 4.58	85.33 ± 4.51	11.33 ± 1.15	52.33 ± 4.51
Group II	140 ± 5.02 **	123.33 ± 5.03 **	68.67 ± 16.17 **	34.67 ± 3.21 **
Group III	98 ± 3.02 *	83.33 ± 0.58 *	24.33 ± 1.53 *	49 ± 5.72
Group IV	97.67 ± 4.73 *	81 ± 1.93 *	16.67 ± 1.15 *	44 ± 1.53
Group V	64.67 ± 1.53 *	80.33 ± 2.52 *	10.67 ± 0.58 *	51.77 ± 5.77

Each value expressed as the mean ± SD (*n* = 3); * Significant at *p*-values < 0.001 as compared to the positive control group. ** Significant at *p*-values < 0.001 as compared to the negative control group.

**Table 7:** Batts–Ludwig histological grading and stage of liver samples obtained from different experimental animal groups.

Group	Grading	Total
Group I	-	0/18
Group II	Interface hepatitis = 1, Confluent necrosis = 1, Lytic necrosis = 2, Portal inflammation = 2	6/18
Group III	Portal inflammation = 1	1/18
Group IV	Interface hepatitis=1, Portal inflammation=1, Focal inflammation = 1	3/18
Group V	Confluent necrosis = 1	1/18

## Data Availability

Not applicable.

## Supplementary Materials

The following are available online at https://www.mdpi.com/1999-4923/13/2/178/s1, Table S1: Experimental domain of the two-level D-optimal screening experimental design of the formulation parameters for the 46 runs and their measured responses, Figure S1: Microscopic photos representing both the inflammatory changes and fatty changes in the experimental animals’ liver samples. A represents the inflammatory change in Group I (a and c), Group II (b and d), Group III (e and g), Group IV (f and h), and Group V (k and i); B represents the fatty change (steatosis) in Group II (a) and Group V (b).
