# Supplementary material for: Comprehensive Study of Atorvastatin Nanostructured Lipid Carriers through Multivariate Conceptualization and Optimization
Source: Pharmaceutics. 2021 Jan 28;13(2):178. doi: 10.3390/pharmaceutics13020178 (PMC7911144; doi:10.3390/pharmaceutics13020178)
Supplement: Supplementary file 1 [file pharmaceutics-13-00178-s001.pdf]

pharmaceutics

MDPI

Supplementary Materials: Comprehensive Study of Atorvastatin Nanostructured Lipid Carriers through Multivariate Conceptualization and Optimization

Heba A. Ghanem, Ali M. Nasr, Tamer H. Hassan, Mahmoud M. Elkhoudary, Reem Alshaman, Abdullah Alattar and Shadeed Gad

Table S1. Experimental domain of two-level D-optimal screening experimental design of the formulation parameters for the 46 runs and their measured responses.

Block	R	A	B	C	D	E	F	G	H	J	K	L	R1	R2	R3
Day 1	1	100	0	100	0	0.5	16,000	5	6	75	No	P188	−1.3 ± 0.20	27.8 ± 0.28	0.4 ± 0.01
Day 1	2	100	0	0	100	0.5	12,000	10	4	25	No	P188	−4.1 ± 0.80	611.3 ± 1.97	0.3 ± 0.02
Day 1	3	0	100	0	100	0.5	12,000	5	4	75	No	T80	−2.7 ± 0.80	59.1 ± 1.51	0.44 ± 0.01
Day 1	4	100	0	0	100	0.5	12,000	10	4	25	No	P188	−3.8 ± 0.30	513.5 ± 6.47	0.26 ± 0.04
Day 1	5	0	100	0	100	0.5	16,000	10	4	25	Yes	T80	−4.2 ± 1.10	20.1 ± 0.11	0.41 ± 0.02
Day 1	6	100	0	100	0	1.5	12,000	10	4	25	Yes	T80	−15.3 ± 0.30	82.1 ± 0.81	0.23 ± 0.03
Day 1	7	0	100	0	100	0.5	16,000	10	6	75	No	P188	−4.4 ± 0.20	31.5 ± 0.11	0.38 ± 0.02
Day 1	8	0	100	100	0	0.5	12,000	10	4	25	No	P188	−1.6 ± 2.80	17.7 ± 0.33	0.45 ± 0.00
Day 1	9	0	100	0	100	0.5	12,000	5	6	25	Yes	P188	0.4 ± 0.20	22.2 ± 0.29	0.48 ± 0.12
Day 1	10	0	100	0	100	1.5	16,000	5	6	25	No	T80	0.3 ± 0.80	15.8 ± 1.13	0.55 ± 0.02
Day 2	11	0	100	100	0	1.5	12,000	5	4	25	No	T80	−9.3 ± 0.40	25.9 ± 0.37	0.45 ± .15
Day 2	12	100	0	0	100	0.5	16,000	5	6	25	No	T80	−1.1 ± 0.20	2168 ± 88.1	0.31 ± 0.05
Day 2	13	0	100	0	100	0.5	16,000	5	6	75	Yes	T80	−0.3 ± 0.30	25.8 ± 0.45	0.9 ± 0.01
Day 2	14	100	0	0	100	1.5	16,000	10	4	75	No	T80	−1.4 ± 0.50	181.4 ± 7.93	0.49 ± 0.06
Day 2	15	100	0	0	100	0.5	12,000	5	4	75	Yes	T80	−15 ± 1.00	682.4 ± 43.71	0.53 ± 0.04
Day 2	16	0	100	0	100	1.5	12,000	10	4	25	No	P188	−12.8 ± 0.40	107.4 ± 0.67	0.5 ± 0.12
Day 2	17	100	0	0	100	0.5	12,000	5	4	75	Yes	T80	-22 ± 0.70	672.7 ± 114.00	0.87 ± 0.11
Day 2	18	100	0	0	100	1.5	12,000	10	6	25	Yes	T80	−6.4 ± 0.70	3919. 7 ± 217.30	0.71 ± 0.01
Day 2	19	0	100	100	0	0.5	16,000	10	4	25	Yes	P188	−1.1 ± 0.40	21.4 ± 0.46	0.43 ± 0.03
Day 2	20	100	0	0	100	1.5	16,000	10	4	75	No	T80	−0.8 ± 0.60	60.3 ± 2.04	0.71 ± 0.02
Day 3	21	0	100	100	0	1.5	12,000	10	4	75	Yes	T80	−26.4 ± 0.40	69.4 ± 0.56	0.47 ± 0.04
Day 3	22	100	0	100	0	1.5	12,000	10	6	25	No	P188	−2.7 ± 0.10	4330.3 ± 3212.00	0.11 ± 0.03
Day 3	23	100	0	100	0	0.5	12,000	10	4	75	Yes	P188	−25.7 ± 1.30	143. 7 ± 3.60	0.49 ± 0.04
Day 3	24	100	0	100	0	1.5	16,000	5	4	25	Yes	P188	−11.8 ± 0.30	165.5 ± 5.30	0.35 ± 0.13
Day 3	25	0	100	100	0	1.5	16,000	5	6	75	No	P188	0.6 ± 0.50	17.3 ± 1.00	0.67 ± 0.02
Day 3	26	0	100	100	0	0.5	12,000	5	6	25	Yes	T80	−0.4 ± 0.50	16.3 ± 0.40	0.47 ± 0.20
Day 3	27	100	0	100	0	1.5	16,000	10	4	75	No	T80	−13.5 ± 0.50	121.8 ± 10.80	0.84 ± 0.01
Day 3	28	100	0	100	0	0.5	16,000	10	6	25	Yes	T80	−10.6 ± 0.50	47.6 ± 1.70	0.35 ± 0.04
Day 4	29	25	75	25	75	0.5	16,000	5	4	25	No	P188	−2.3 ± 0.70	23.5 ± 0.20	0.41 ± 0.11
Day 4	30	0	100	100	0	1.5	12,000	10	6	25	No	P188	−0.6 ± 0.40	17 ± 0.80	0.54 ± 0.01
Day 4	31	0	100	0	100	1.5	12,000	10	6	75	Yes	T80	−1.9 ± 0.30	83.8 ± 1.20	0.7 ± 0.03
Day 4	32	0	100	100	0	0.5	12,000	5	6	75	Yes	P188	−0.3 ± 0.50	17.4 ± 0.30	0.5 ± 0.26
Day 4	33	100	0	0	100	1.5	16,000	5	4	25	Yes	P188	−19.6 ± 1.60	6983.3 ± 2014.8	0.55 ± 0.00
Day 4	34	100	0	100	0	1.5	12,000	5	6	75	Yes	T80	−21.3 ± 0.70	122.2 ± 1.30	0.51 ± 0.00
Day 4	35	0	100	100	0	0.5	16,000	5	4	75	No	T80	−0.6 ± 0.40	15.3 ± 0.30	0.44 ± 0.01
Day 4	36	0	100	100	0	1.5	16,000	10	6	25	Yes	T80	−3.6 ± 0.70	22.7 ± 0.70	0.45 ± 0.00
Day 5	37	100	0	100	0	0.5	12,000	5	4	25	No	T80	−2.1 ± 0.40	195.2 ± 11.50	0.21 ± 0.12
Day 5	38	100	0	0	100	0.5	16,000	10	6	75	Yes	P 188	0.4 ± 0.70	424.7 ± 43.10	0.81 ± 0.12
Day 5	39	0	100	0	100	1.5	16,000	5	4	75	Yes	P 188	−9.1 ± 0.40	387.1 ± 36.70	0.92 ± 0.12
Day 5	40	100	0	0	100	0.5	16,000	10	6	75	Yes	P188	−0.5 ± 0.10	517.9 ± 69.70	0.92 ± 0.12
Day 5	41	25	75	0	100	0.5	12,000	10	6	25	No	T80	−0.5±0.10	23.4±1.60	0.58 ± 0.04
Day 5	42	100	0	0	100	1.5	12,000	5	6	75	No	P 188	−1±1.50	27.9±0.30	0.8 ± 0.04
Day 5	43	75	25	100	0	1.5	16,000	10	6	75	Yes	P 188	−13.7±0.60	104.7±1.10	0.48 ± 0.05
Day 5	44	100	0	0	100	1.5	12,000	5	6	75	No	P188	0.2±0.50	580.1±99.10	0.95 ± 0.05
Day 5	45	0	100	100	0	0.5	12,000	10	6	75	No	T80	−15.3±0.50	171±6.90	0.52 ± 0.05
Day 5	46	0	100	100	0	1.5	12,000	5	4	75	Yes	P188	−12.5±0.40	84.4±0.60	0.49 ± 0.01

Note: P188: Poloxamer 188; T80: Tween 80; R: run number; A: Labrasol (%); B: Oleic acid (%); C: Gelucire 43/01 (%); D: Stearic acid (%); E: Surfactant concentration (%); F: Homogenization speed (rpm); G: Sonication time (min); H: Total lipid (%); J: Solid/Liquid lipid ratio; K: Lecithin and L: Surfactant type; R1: zeta potential (mV); R2: Particle size (nm), and R3: polydispersity index

A: Inflammatory Change	
Group I	Group II
a) 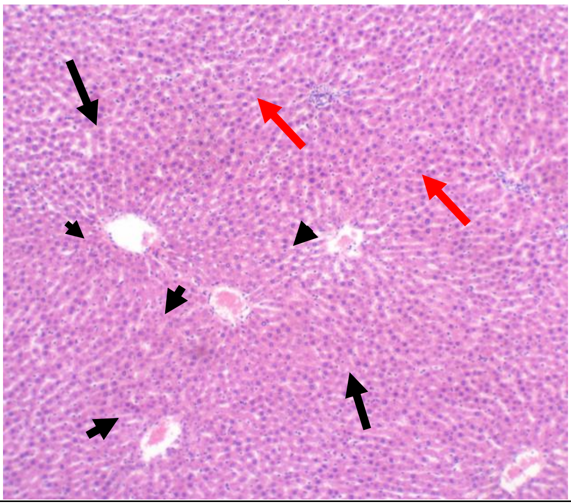	b) 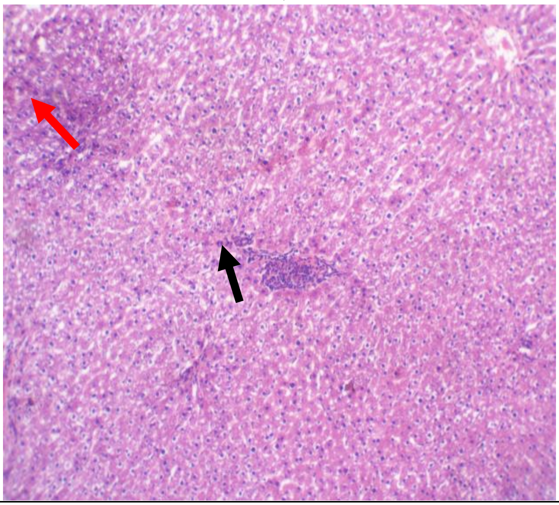
Liver architecture is preserved. Plates of hepatocytes (Black arrows) radiating from central vein (Arrow heads), with uniform portal tracts (Red arrows) (H&E, 10×)	Liver architecture is preserved, but there are foci of portal inflammation (Black arrow) and focal confluent necrosis (Red arrow) (H&E, 10×)
c) 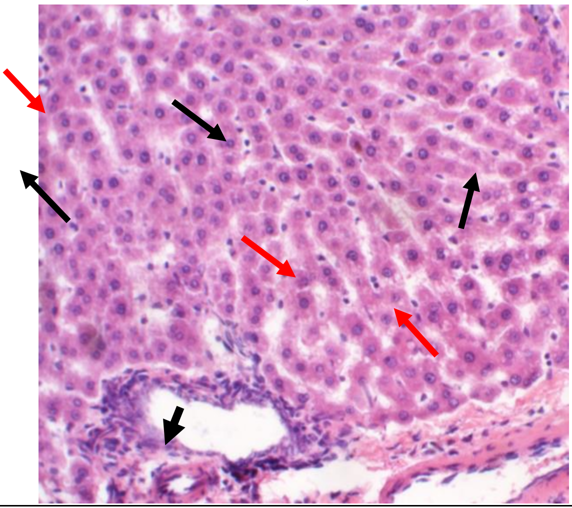	d) 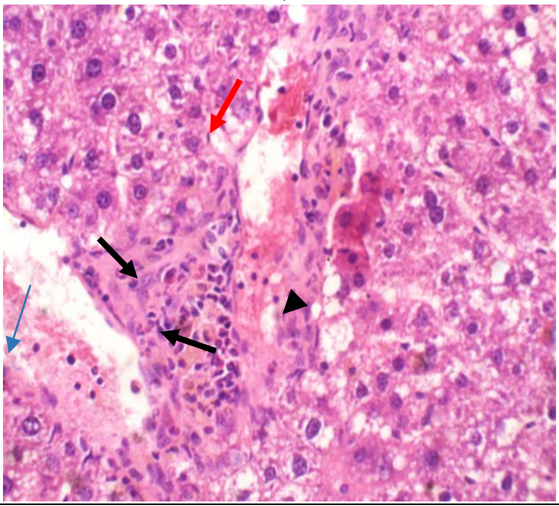
Uniform hepatocyte plates (Black arrows), patent sinusoids (Red arrows), and uniform portal area (Arrowhead) (H&E, 40×)	Higher magnification of a focus showing lytic necrosis (Black arrow), congested sinusoids (Red arrow), and a congested central vein (Blue arrow) (H&E, 40×)

Group III	Group IV
**e)** 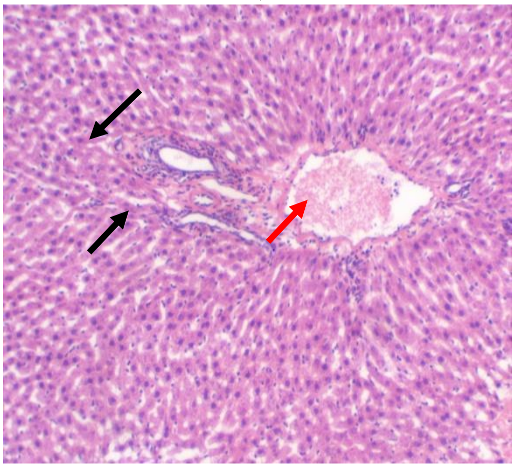	**f)** 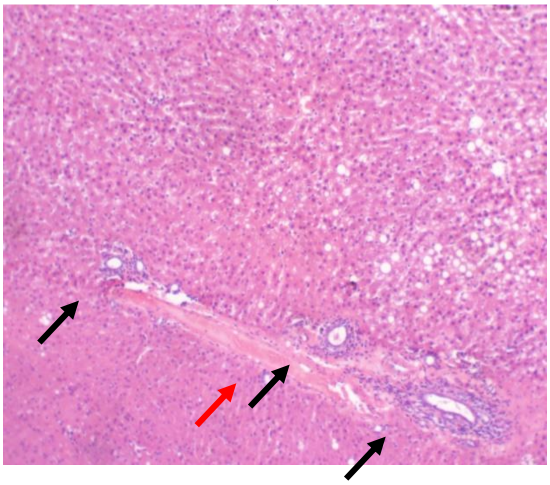
**There is mild expansion of a few portal tracts with lymphocytic infiltrate (Black arrows) with a congested vessel (Red arrow) (H&E, 10×)**	**There is mild expansion of a few portal tracts with lymphocytic infiltrate (Black arrows) with a congested vessel (Red arrow) (H&E, 10×)**
**g)** 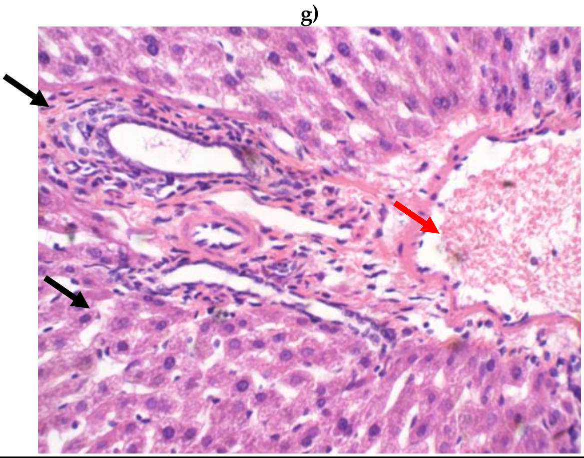	**h)** 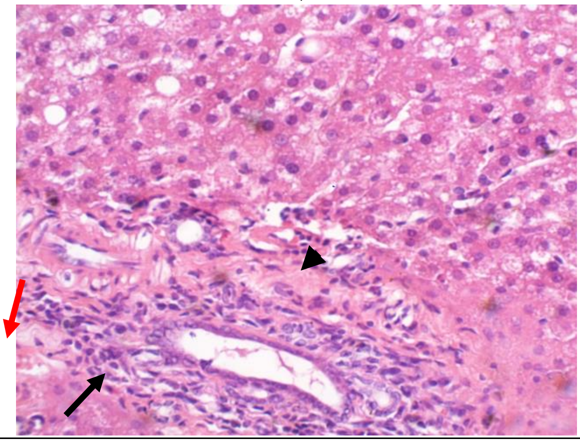
**Higher magnification of the portal area to highlight mild portal tract inflammation (Black arrow) and a congested vessel (Red arrow) (H&E, 20×)**	**Moderate portal tract inflammation (Black arrow), mild interface hepatitis (Red arrow), and a single focus of focal inflammation (Arrowhead) (H&E, 40×)**

**Group V**	
**k)**	**i)**
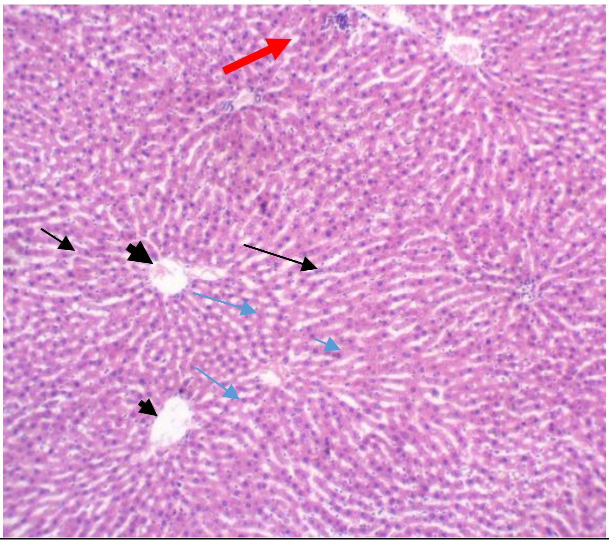	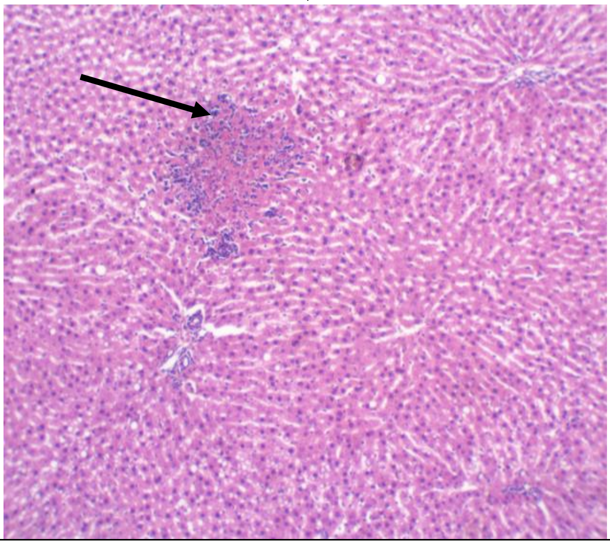
**Liver architecture is preserved. Plates of hepatocytes (Black arrows) radiating from the central vein (Arrow heads), with uniform portal tracts (Red arrows) and patent sinusoids (Blue arrows) (H&E, 10×)**	**A single focus of confluent necrosis (Black arrow) is identified (H&E, 10×)**
**B: Fatty Change**	
**Group II**	**Group V**
**a)**	**b)**
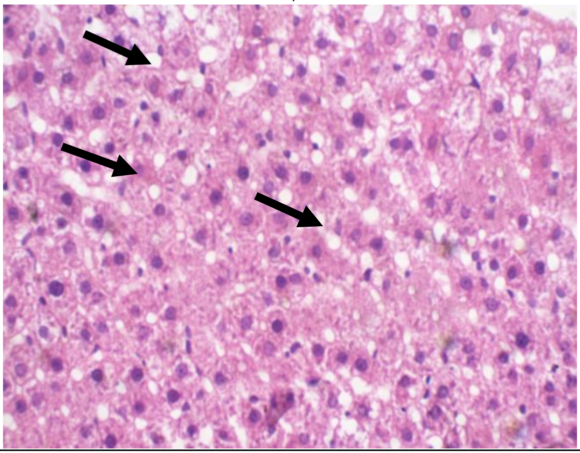	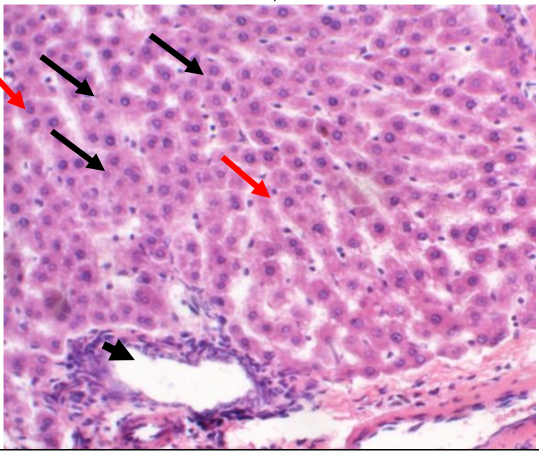
**Majority of hepatocytes show accumulation of triglycerides (steatosis). The black arrow highlights fatty changes in hepatocytes (H&E, 100×)**	**Uniform hepatocyte plates (Black arrows), patent sinusoids (Red arrows), and uniform portal area (Arrowhead) (H&E, 40×)**

Figure S1. Microscopic photos representing both the inflammatory changes and fatty changes in the experimental animals' liver samples. A represents the inflammatory change in Group I (a and c), Group II (b and d), Group III (e and g), Group IV (f and h), and Group V (k and i); B represents the fatty change (steatosis) in Group II (a) and Group V (b). Note: H&E: hematoxylin/eosin.
